# Integrative physiology and systems biology: reductionism, emergence and causality

**DOI:** 10.1186/2046-7648-2-9

**Published:** 2013-03-22

**Authors:** Michael P W Grocott

**Affiliations:** 1 Integrative Physiology and Critical Illness Group, Division of Clinical and Experimental Science, Faculty of Medicine, University of Southampton, University Road, Southampton, SO17 1BJ, UK; 2 Anaesthesia and Critical Care Research Unit, University Hospital Southampton, NHS Foundation Trust, CE.93 Mailpoint 24, E-Level Centre Block, Tremona Road, Southampton, SO16 6YD, UK; 3 The Royal College of Anaesthetists, Churchill House 35 Red Lion Square, London, WC1R 4SG, UK

## 

*Systems biology…is about putting together rather than taking apart, integration rather than reduction. It requires that we develop ways of thinking about integration that are as rigorous as our reductionist programmes, but different.... It means changing our philosophy, in the full sense of the term.* Dennis Noble, 2006 [1].

Francis Crick's ‘Central Dogma’, which states that DNA encodes mRNA (transcription) and mRNA encodes protein (translation), has provided a conceptual basis for the biomedical sciences for more than 50 years [2]. However, the limitations of this framework are increasingly clear: inherited genetic code is a necessary, but not sufficient, explanation of how and why cells within a living organism behave as they do. The critical role of epigenetic change during life in determining the differentiation status of each cell has added a new dimension to our understanding of the relationship between genotype and phenotype. The heritability of some of these changes through mechanisms independent of alterations in the DNA sequence of base pairs has equally profound implications. Perhaps even more importantly, the assumption of linear causality (from gene via transcript to protein and thence function) implicit in Crick’s Central Dogma may in itself be flawed. Emergence, defined as ‘the arising of novel and coherent structures, patterns and properties during the process of self-organization in complex systems’ [3], is arguably a defining characteristic of higher organisms: the Cartesian premise that the whole is no more than the sum of its parts is hard to defend when considering the physiology of complex eukaryotes. Cellular differentiation and the development of specific physiological functions are clearly determinist processes; the extraordinarily consistent development of intricate phenotypes in humans and other highly evolved species cannot conceivably be the result simply of stochastic processes. However, the nature of these processes may not be best explored through reductionist experimental approaches. Indeed, such approaches may, by their nature, fail to identify or account for emergent phenomena. In the reductionist paradigm, the multiple interacting systems of intact human physiology obscure the basic mechanisms underpinning phenotypic variation: they are the physiological ‘noise’ obscuring the biochemical signal. Conversely, the integrative paradigm views these systems and the emergent phenomena that result from them as providing the more relevant signal. To quote from Anderson, ‘The ability to reduce everything to simple fundamental laws does not imply the ability to start from those laws and reconstruct the universe. The constructionist hypothesis breaks down when confronted with the twin difficulties of scale and complexity. At each level of complexity entirely new properties appear. Psychology is not applied biology, nor is biology applied chemistry. We can now see that the whole becomes not merely more, but very different from the sum of its parts’ [4].

The speed and accuracy of biochemical analyses have been transformed in the years since DNA was first characterised as the biochemical substrate of evolution by Crick and Watson. Led by advances in genetic sequencing, the *omics* disciplines have opened a window on the study of biological function (biochemistry and physiology) that is arguably comparable to the revolution in the study of biological form (anatomy) that occurred in the sixteenth and seventeenth centuries with the advent of human cadaver dissection and light microscopy. Whole genome sequencing and high throughput transcriptomic, proteomic and metabalomic analyses are becoming commonplace. At the same time, the explosion in analytical power consequent upon the consistent increases in processing power achieved by modern ‘supercomputers’ is enabling previously inconceivable feats of computational biology. This processing power can be harnessed to explore the multiple hierarchical interrelationships within complex physiological systems and for iterative biological model generation: experimentation *in silico*. An additional benefit of this analytical power is the capacity to examine temporal patterns of variability in putative physiological signals (rather than the absolute values of specific variables). Previously, we have sought average data to identify ‘representative’ values. Now, the pattern of variability of the signal over time (time-series data) is recognised as a signal in itself. It seems likely that the pattern of variation over time exhibited by many physiological variables is a reflection of the robustness of the underlying homeostatic control systems, and that loss of this variability is a sign of dysfunction and ill health. For example, loss of heart rate variability is recognised to be a risk factor for early death following myocardial infarction. It may be that maintenance of complex patterns of variability is a defining feature of beneficial adaptation to environmental stressors; conversely, loss of such variability may be a signal of maladaptation.

This month in *Extreme Physiology & Medicine*, Lindsay Edwards and Ines Thiele review the application of systems biology methods to the study of human integrative physiology, with particular focus on applications relating to conditions of environmental stress [5]. Edwards and Thiele define systems biology as ‘an iterative process of computational model-building and experimental model-revision with the aim of understanding or simulating complex biological systems’. Further, they highlight the limited number of physiologically relevant perturbations that are ethically acceptable in humans and highlight the value of environmental and exertional stressors in this context.

Whether there is truly a distinction between systems biology and integrative physiology is unclear [6]. Both disciplines focus on the form and function of cells and cellular systems through the study of physical and chemical phenomena. As has been commented previously, it is only the amount of data, and the rate of its accumulation, that distinguishes one from the other [7]. That is not to say that systems biology will not yield new insights. The application of the tools and techniques of computational biology to mega data sets incorporating *omics* readouts and high-resolution phenotypic metadata may transform our understanding of phenomena with causation at multiple hierarchical levels within the cell and organism as well as identifying the key determinants of the development of emergent phenomena. Such insights may also fundamentally alter our understanding of causality in physiological systems and thereby shape our views of the very nature of integrative physiology.

## Competing interests

The author declares that he has no competing interests.
